# Taxonomic structure in a set of abstract concepts

**DOI:** 10.3389/fpsyg.2023.1278744

**Published:** 2024-01-04

**Authors:** Andrew S. Persichetti, Jiayu Shao, Joseph M. Denning, Stephen J. Gotts, Alex Martin

**Affiliations:** ^1^Section on Cognitive Neuropsychology, Laboratory of Brain and Cognition, National Institute of Mental Health, National Institutes of Health, Bethesda, ML, United States; ^2^Department of Psychology, University of California Los Angeles, Los Angeles, CA, United States

**Keywords:** categories, abstract words, semantic knowledge, concepts, automatic semantic priming

## Abstract

A large portion of human knowledge comprises “abstract” concepts that lack readily perceivable properties (e.g., “love” and “justice”). Since abstract concepts lack such properties, they have historically been treated as an undifferentiated category of knowledge in the psychology and neuropsychology literatures. More recently, the categorical structure of abstract concepts is often explored using paradigms that ask participants to make explicit judgments about a set of concepts along dimensions that are predetermined by the experimenter. Such methods require the experimenter to select dimensions that are relevant to the concepts and further that people make explicit judgments that accurately reflect their mental representations. We bypassed these requirements by collecting two large sets of non-verbal and implicit judgments about which dimensions are relevant to the similarity between pairs of 50 abstract nouns to determine the representational space of the concepts. We then identified categories within the representational space using a clustering procedure that required categories to replicate across two independent data sets. In a separate experiment, we used automatic semantic priming to further validate the categories and to show that they are an improvement over categories that were defined within the same set of abstract concepts using explicit ratings along predetermined dimensions. These results demonstrate that abstract concepts can be characterized beyond their negative relation to concrete concepts and that categories of abstract concepts can be defined without using *a priori* dimensions for the concepts or explicit judgments from participants.

## Introduction

Storing knowledge about the world as concepts and organizing those concepts into categories is a core feature of human cognition (Rosch, 1973, 1975). Categorization allows us to generalize knowledge across different experiences and to retain seemingly infinite facts about things in the world. A common framework for studying how categories are represented in the mind is to assume that they are comprised of concrete (e.g., “dog,” “hammer,” “carrot”) and abstract (e.g., “love,” “justice,” “intelligence”) concepts (e.g., Schwanenflugel and Shoben, 1983; Paivio, 1991; but also see, Troche et al., 2017; Löhr, 2023). Whether concrete and abstract concepts form a dichotomy or are represented along a unified and continuous space, there is a wealth of evidence that concrete concepts are acquired and processed more easily than abstract concepts – the so-called “concreteness effect” (Gilhooly and Logie, 1980; Bleasdale, 1987; Schwanenflugel et al., 1988; Walker and Hulme, 1999; Allen and Hulme, 2006). Furthermore, prior behavioral studies have demonstrated that concrete categories like “animals,” “tools,” and “food” are represented based on shared physical and functional properties among the category members (Cree and McRae, 2003) – i.e., properties that can be easily referenced by ostension. Since concrete concepts share perceivable and functional properties that can be easily referenced, their category structure is often intuitive and apparent. By contrast, it is thought that the category structure of abstract concepts is not so intuitive because, by definition, they lack readily perceivable properties (Paivio, 1991; Borghi et al., 2017; Desai et al., 2018) and therefore the domain of abstract concepts has often been treated as an undifferentiated category in the both the psychology and neuropsychology literatures (Warrington, 1975; Schwanenflugel and Shoben, 1983; Riddoch et al., 1988; Paivio, 1991; Plaut and Shallice, 1993; Breedin et al., 1994; Franklin et al., 1995).

More recently, many experiments have mapped the representational space of abstract concepts using methods that rely on participants making explicit judgments about concept properties and category membership based on featural dimensions and categories that are predetermined by the experimenter (e.g., McRae et al., 2005; Crutch et al., 2013; Devereux et al., 2014; Villani et al., 2019; Lynott et al., 2020; Banks and Connell, 2023). However, such experimental paradigms might not be well-suited to mapping the representational space of abstract concepts because the featural dimensions of the space and the categories within it may not be known *a priori* or might be hard to articulate. In addition, these experiments ask people to make explicit verbal judgments about concepts that accurately reflect their mental representations. Prior findings suggest that these requirements might introduce unnecessary bias into the experiment. For example, when asked to freely list the semantic features of concepts, people generate only a small subset of relevant features (Plaut and Shallice, 1993; McRae et al., 2005). On the other hand, experiments that ask participants to rate concepts along featural dimensions might not be adequate for mapping the representational space of abstract concepts because the dimensions will be limited in number and thus biased by the experimenter’s choice of which dimensions to include in the experiment. The goal of the current study is to use an analytic approach that does not rely on *a priori* featural dimensions or explicit judgments from participants to identify behaviorally relevant categories in a set of abstract words. To do so, required us to choose a relatively small set of 50 abstract nouns so we could exhaustively sample the representational space of the concepts with tens of thousands of similarity judgments using an odd-one-out triad task (Experiment 1) and subsequently collect over a thousand trials during an automatic semantic priming in each participant (Experiment 2).

In Experiment 1, we used an odd-one-out similarity task, in which participants chose which of three abstract nouns was least like the other two across many trials, to determine the representational space of 50 abstract nouns (Hebart et al., 2020). Using this approach provides a non-verbal and implicit judgment about which dimensions are most relevant to the similarity between pairs of words across varying contexts provided by the third word in each trial, and thus makes it possible to capture data-driven dimensions that might otherwise be missed. We then used the InfoMap clustering algorithm to define categories within the representational space (Rosvall and Bergstrom, 2008, 2011). In Experiment 2, we used automatic semantic priming to confirm that the categories found in Experiment 1 reflect intrinsic semantic categories rather than deliberative *ad hoc* categories (Barsalou, 1983) and to test whether reshuffling the words into categories based on explicit ratings along predetermined dimensions would also produce priming (Neely, 1977, 1991; Sperber et al., 1979; Lucas, 2000; McNamara, 2005).

### Experiment 1

In the first experiment, we collected two sets of similarity judgments, each comprising tens of thousands of implicit judgments of the similarity between 50 abstract nouns on Amazon’s Mechanical Turk (M Turk). We then constructed two independent similarity matrices from those data and searched for clusters that replicated across them. The goal was to identify stable categories among the set of abstract words.

## Methods

### Participants

Eleven hundred and four individuals (mean (SD) age, 39.8 (11.9); 436 females) participated in the experiment on M Turk. Participants were required to be high school graduates, native English speakers, and live within the United States. These participants gave informed consent under the National Institutes of Health (NIH) Institutional Review Board-approved protocol number 000589 and were compensated financially for their time.

### Experimental design

We created a set of 50 abstract nouns by randomly choosing common English words that were at least one standard deviation below the mean concreteness rating in the Abstract Conceptual Feature (ACF) database (Crutch et al., 2013; Troche et al., 2017). There were 169 words that were at least one standard deviation below the mean concreteness rating. Therefore, our method of randomly selecting words was to list and number those words and then randomly draw 50 numbers from the range of 1–169 using the *randperm* function in Matlab. We then posted an odd-one-out triad task on M Turk to obtain similarity judgements for these 50 abstract nouns. On each trial of the experiment, three words were presented in random order in a horizontal row under the instruction to “click on the odd-one out.” The participants used a computer mouse to click on the chosen word, which was followed by a blank screen for 500 milliseconds (ms), then the next trial. Participants were given as much time as needed to make their choice. The set of all possible triads (19,600 trials) was randomly ordered and divided into 980 unique blocks, each with 20 triad trials. To ensure that participants were attentive during each block, we included four catch trials in which participants saw the words “PLUS,” “MINUS,” and “EQUAL” randomly ordered in a horizontal line under the instruction to “click on EQUAL.” Thus, there were 24 trials in each block. Individuals were allowed to participate in a maximum of five blocks. Before starting the experiment, participants were presented with instructions and an example triad that included words that were not used in the experiment. After collecting the full set of triads, we ran the same experiment again to obtain a second set of similarity judgements from a separate group of participants. Before posting the second set of triads, we randomized the order of triads before dividing them into blocks and excluded individuals that participated in the first posting. We excluded blocks in which the participant either (1) failed to supply the correct answer on a catch trial; (2) had an average trial response time of less than 500 ms; (3) or selected the same response on every trial. In the event of a rejected block, the individual was excluded from further participation in the experiment and the block was posted again. A total of 276 subjects were eliminated based on the criteria described above. In total, we used the data from 828 participants to collect two full sets of 19,600 trials each.

### Data analysis

Based on the results of the odd-one-out triad task, we created two separate 50×50 similarity matrices (one for each full set of triads) by calculating the proportion of trials in which a given word pair was kept together across all triads containing that word pair, then calculating Euclidean distances between the words. The independent similarity matrices were strongly correlated (r = 0.91). We then clustered the abstract nouns using the similarity matrices in the following way. First, the real-valued similarity matrices were thresholded into binary (0 or 1) undirected matrices at a range of threshold values (top %: 50, 60, 70, 80, 85, 90, 95, 99, 99.5). The thresholded matrices were then clustered using the InfoMap algorithm to form optimal two-level partitions (i.e., the optimal solution found on 100 searches). Across the range of thresholds, we found optimal agreement of number of clusters found in the two matrices and the percentage of words clustered across the matrices at the 90% threshold. We then constrained the clusters that replicated across the two matrices to contain at least four words to ensure that each cluster was stable and potentially interpretable. Since the two similarity matrices were strongly correlated, we averaged them together before performing hierarchical clustering for greater precision and stability.

## Results

**Figure 1:**
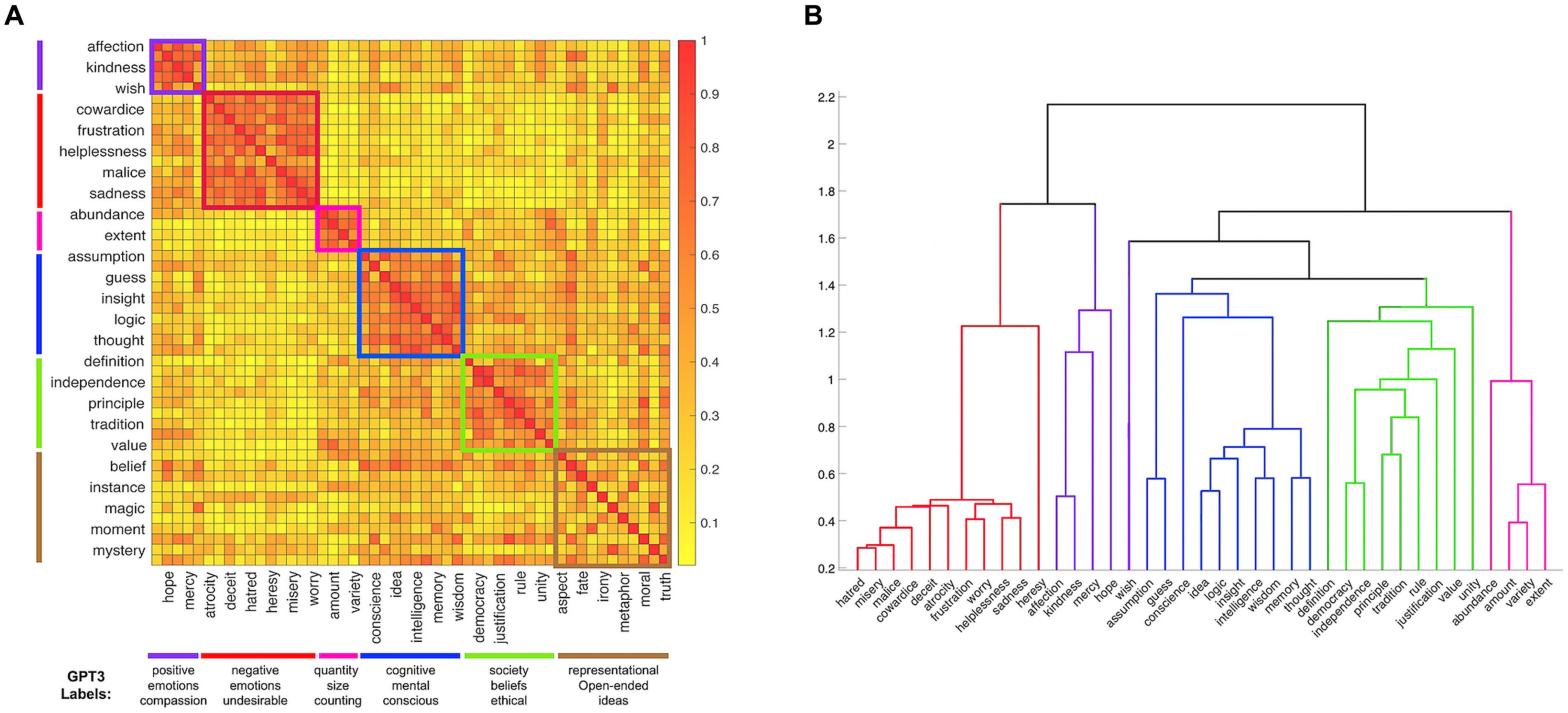
**(A)** The similarity matrix for the 50 abstract nouns based on the odd-one-out triad task. For readability, the odd rows and even columns of the matrix are labeled. The five categories and the cluster of unsorted words are color coded both by squares along the diagonal of the matrix and lines on the outer sides of the matrix. The top three labels generated by the GPT3 model are listed at the bottom of the figure, beneath each category. **(B)** The dendrogram showing the hierarchical organization of 39 abstract nouns into basic- and superordinate- level categories (the 11 words that were left unsorted are not included). The branches are color coded using the same scheme used in Figure 1A.

The clustering procedure separated 39 abstract nouns into five replicable clusters that each included at least four words, while the remaining 11 words were left unsorted based on the conservative inclusion criteria described above (Figure 1A). Our procedure clustered the abstract nouns into categories that seem to capture meaningful dimensions of the stimuli. To generate category labels, we asked OpenAI’s text-davinci-003 GPT3 model (Brown et al., 2020) to list properties that are common across the words from each category [The exact prompt was “List five common properties of the following words: (list of words from the category)].” The top three labels for each category (and the cluster unsorted words) are listed at the bottom of Figure 1A. Based on these labels, the categories can be described as relating to positive emotions, negative emotions, quantities, cognitive terms, and social terms, respectively. By contrast, the cluster of unsorted words was labeled with vague terms, such as “representational” and “open-ended.”

Next, we asked whether the 39 abstract nouns that were clustered into five categories were further nested in a hierarchy comparable to the well-established organization of concrete nouns into subordinate, basic, and superordinate levels, Rosch (1973). A hierarchical clustering analysis based on the Euclidean distances between the categorized words indeed resulted in a three-level dendrogram with the individual words on the bottom; the words grouped into five categories at the middle level (positive and negative emotions, respectively, cognitive, social, and quantitative categories); and three groupings at the upper level. At the upper level of the hierarchy, one branch was affect-related, grouping the positive and negative emotions together, another branch grouped the cognition-related category with the social category, and the third branch was comprised the quantity-related category only (Figure 1B). Thus, like the category structure of concrete nouns, the category structure in our set of abstract nouns is hierarchical.

While our method uncovers a category structure within the space of abstract nouns that was replicated across two data sets, we wanted to compare it to more commonly used methods of investigating the representational space of abstract nouns. To do so, we re-clustered the data using k-means to cluster the 50 abstract nouns based on Euclidean distances derived from explicit feature ratings from the ACF database. We found that the k = 5 cluster solution explained ~80% of the variance in these ratings. However, the clusters do not seem to have a reasonable organizing principle (Supplementary Figure 1). When we asked the GPT3 model to list properties that are common across the words from each of the five clusters, the labels were vague, often referring to the fact that each cluster contains abstract nouns from the English language (Supplementary Figure 1). Taken together, our method of collecting non-verbal and implicit judgments about the dimensional space of abstract words and using a rigorous clustering method revealed a replicable and intuitive category structure within our set of abstract nouns. Furthermore, our method seems to be an improvement over commonly used methods of characterizing the category structure of concepts based on participants making explicit judgments about concept properties along featural dimensions that are predetermined by the experimenter.

### Experiment 2

The results from Experiment 1 demonstrated that using our method of collecting similarity judgments with a triad task to identify categories in a set of abstract nouns provides more reasonable categories compared to commonly used methods that collect explicit judgments about *a priori* semantic features of words. However, since there was no time limit for choosing the odd-one-out of a triad of abstract words, it could be argued that our triad task elicited a controlled and strategic process, as opposed to being implicit and automatic in nature. As a result, the triad task may have produced arbitrary categories rather than the intrinsic semantic categories that we intended to uncover (Barsalou, 1983). To address this potential difficulty, we sought to demonstrate that the five categories identified in Experiment 1 could be activated automatically by using an automatic semantic priming paradigm.

The assumption underlying automatic semantic priming is that the presentation of a word automatically activates its semantic representation in long-term memory and this activation partially includes semantically related concepts due to overlap of shared features, thus increasing the accessibility of those related concepts. Hence, when individuals are required to make a semantic judgment about a word (the probe), they are faster to respond when the probe word is immediately preceded by a semantically related word (the semantic prime) relative to when it is preceded by a semantically unrelated word. Critically, it has been shown that if the words are presented with a very brief time between them (i.e., a stimulus onset of asynchrony – SOA – of 250 ms), then priming reflects automatic activation of semantically related concepts from the same category (e.g., “bulldog” and “poodle”), instead of controlled and conscious associations between related concepts from different categories (e.g., “bulldog” and “leash”) that can occur with longer SOAs (Neely, 1977; Lucas, 2000). Thus, we used a 250 ms SOA to test the intrinsic nature of the categories found in Experiment 1. If this was indeed the case, then participants should be significantly faster to respond to words when they are preceded by words from the same category relative to when they are preceded by words from a different category.

## Methods

### Participants

Twenty-five individuals (mean (SD) age, 27.5 (8.9); 15 females) participated in the experiment in-person at the NIH. These participants gave informed consent under a National Institutes of Health Institutional Review Board-approved protocol (93-M-0170, clinical trial #NCT00001360). An additional 108 individuals (mean (SD) age, 43.1 (9.7); 43 females) participated on M Turk. These participants gave informed consent under the National Institutes of Health Institutional Review Board-approved protocol number 000589. All participants were high school graduates, native English speakers, lived within the United States, and were compensated financially for their time.

### Experimental design

**Figure 2:**
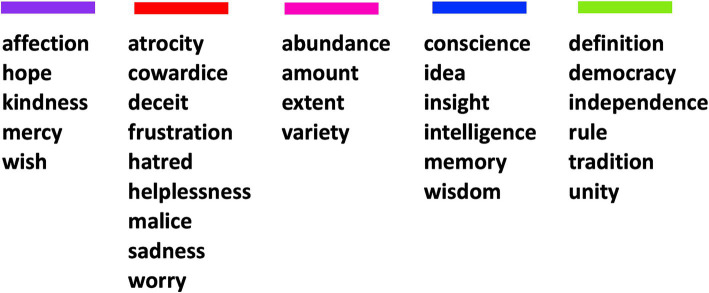
The 30 abstract words used in the automatic semantic priming experiment, separated (and color coded) by the categories identified in Experiment 1.

Prior to generating the word pairs used in the experiment, we chose to exclude nine of the categorized abstract words so that we could present enough trials for evaluating priming, without overtaxing the participants. Thus, we selected 30 words from the categories identified in Experiment 1 to use as stimuli. At least four words were selected from each of the five categories (Figure 2). No other criteria were used to decide which words were excluded. We also selected 30 common concrete nouns from five categories (fruit, four-legged animals, dwellings, tools, and birds) included in the Connecticut Category Norms database (Battig and Montague, 1969) as a control condition (Supplementary Figure 2). We chose these five categories because they should be common to English speakers and they each had enough words that were frequently listed by respondents in the Connecticut Category Norms database so that we could exclude words that were ambiguous or polysemous – e.g., we excluded the word “orange” from the fruit category because it is also commonly used to refer to a color. We compared the magnitude of the automatic semantic priming effects elicited by these concrete words with the priming effects found across the abstract categories.

Semantic priming was tested using a 250 ms SOA. During each trial, a prime word was presented for 100 ms, followed by a 50 ms mask, and 100 ms blank screen, then a probe word for 250 ms, and finally a blank screen for 1,000 ms. The participants were asked to respond using the keyboard whether the probe word was abstract or concrete, by pressing either the “f” or “j” key, respectively. The mapping of response to key press was alternated across participants. Participants were able to respond as soon as the probe word appeared on the screen. Reaction times (RTs) were recorded as the time between the onset of the probe word and the button response on each trial. Before starting the experiment, participants completed 10 practice trials. During the experiment, each participant was presented with 1,312 trials so that every possible prime-probe word pair from within each category was shown twice along with an equal number of between-category word pairs. Participants also saw an equal number of trials in which the prime was an abstract noun while the probe was concrete and vice versa (mixed-word-type trials). Thus, there were an equal number of trials in which the correct response was ‘abstract’ or ‘concrete’ across the experiment. The trials were randomly shuffled and separated into eight runs for each participant. Each run lasted 6 min and 22 s.

### Data analysis

The data were processed using Matlab. For each participant, trials with incorrect responses or RTs that were two and a half standard deviations above or below the mean were excluded from further analyses. Participants were excluded from the analysis if their accuracy was less than 80% across all trials or the average RT across the experiment was two and a half standard deviations above or below the group mean. Across participants, accuracy was very high (94.7%) and there was not a significant difference in accuracy across within- and between-category trials for abstract or concrete words (both *t*’s < 1.57, both *p*’s > 0.10). None of the 25 participants were excluded from the analysis. The RTs for all within- and between- category trials were separately averaged for the abstract and concrete words. The mixed-word-type trials were not analyzed further. Repeated-measures ANOVAs and planned paired-sample t-tests were used to compare the within- and between- category RTs for abstract and concrete words across participants.

### M Turk replication

One-hundred and eight people were recruited from the M Turk Master Subject pool to complete the same automatic semantic priming task as described above. Reaction time data were collected and analyzed using the same procedure as the in-person semantic priming task described above. After data processing, two participants were excluded from the analysis: one because the average RT was above two and a half standard deviations from the group mean and the other for chance-level accuracy.

## Results

**Figure 3:**
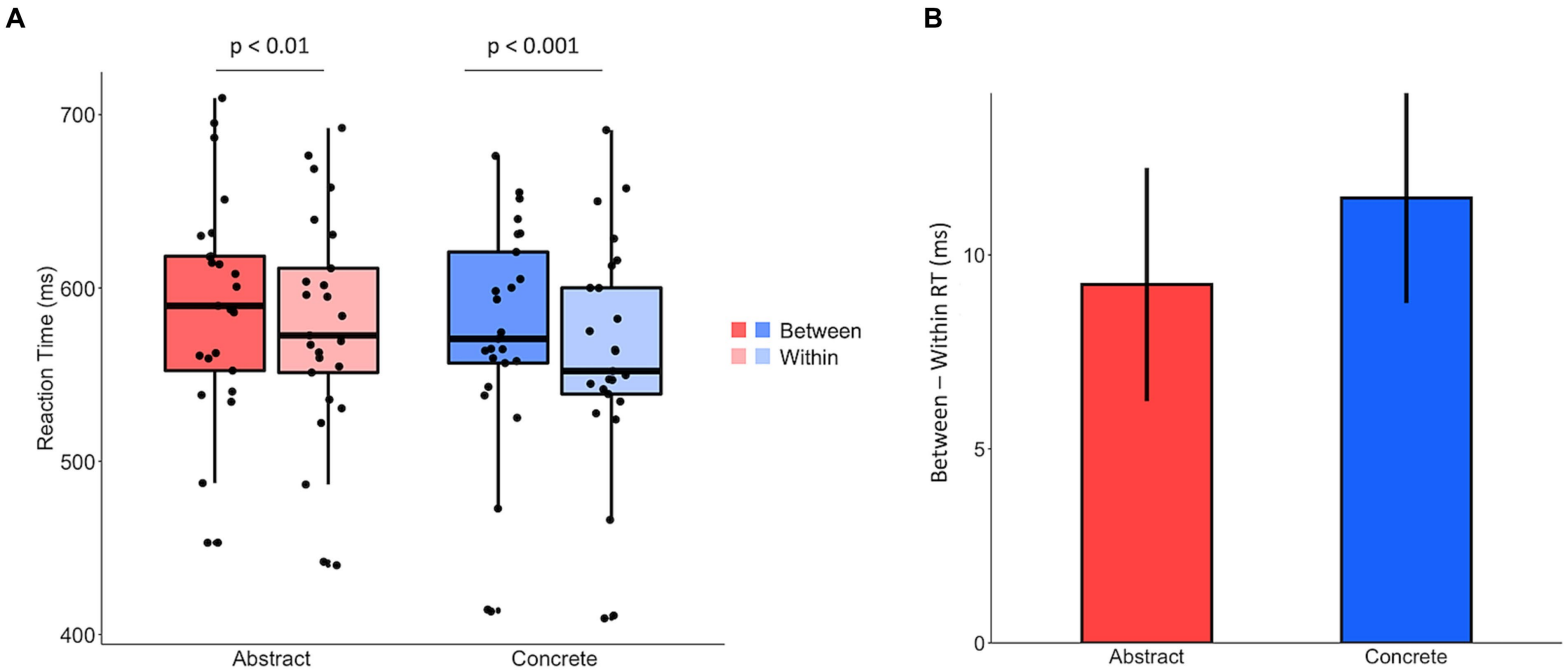
**(A)** The averaged RTs for between- and within- category trials for abstract and concrete nouns, respectively. **(B)** The data from **(A)** displayed as difference scores, so the magnitude of priming for abstract and concrete nouns is more apparent. The difference scores were calculated by subtracting the average within-category RT from the average between-category RT in each participant and then averaged across the group. The magnitude of priming was not significantly different between abstract and concrete nouns (*p* = 0.54).

Paired-sample t-tests revealed significant priming effects (i.e., greater between- than within- category RTs) for both abstract (*t*_(24)_ = 3.07, *p* < 0.01) and concrete (*t*_(24)_ = 4.23, *p* < 0.001) words (Figure 3A). Furthermore, a 2 (Abstract, Concrete) × 2 (Within, Between) repeated-measures ANOVA revealed a main effect of word type (*F*_(1,24)_ = 11.64, p < 0.01), but not a significant interaction (*F*_(1,24)_ = 0.385, *p* = 0.54). These results demonstrate that while participants were faster to respond to concrete words, the magnitude of priming was not significantly different for abstract and concrete nouns (Figure 3B). To ensure that the priming effect for the abstract categories was robust, we replicated the result in 106 participants on M Turk. Paired-sample t-tests again found significant priming effects for both abstract (*t*_(105)_ = 2.07, *p* < 0.05) and concrete (*t*_(105)_ = 7.33, *p* < 0.001) words.

**Figure 4:**
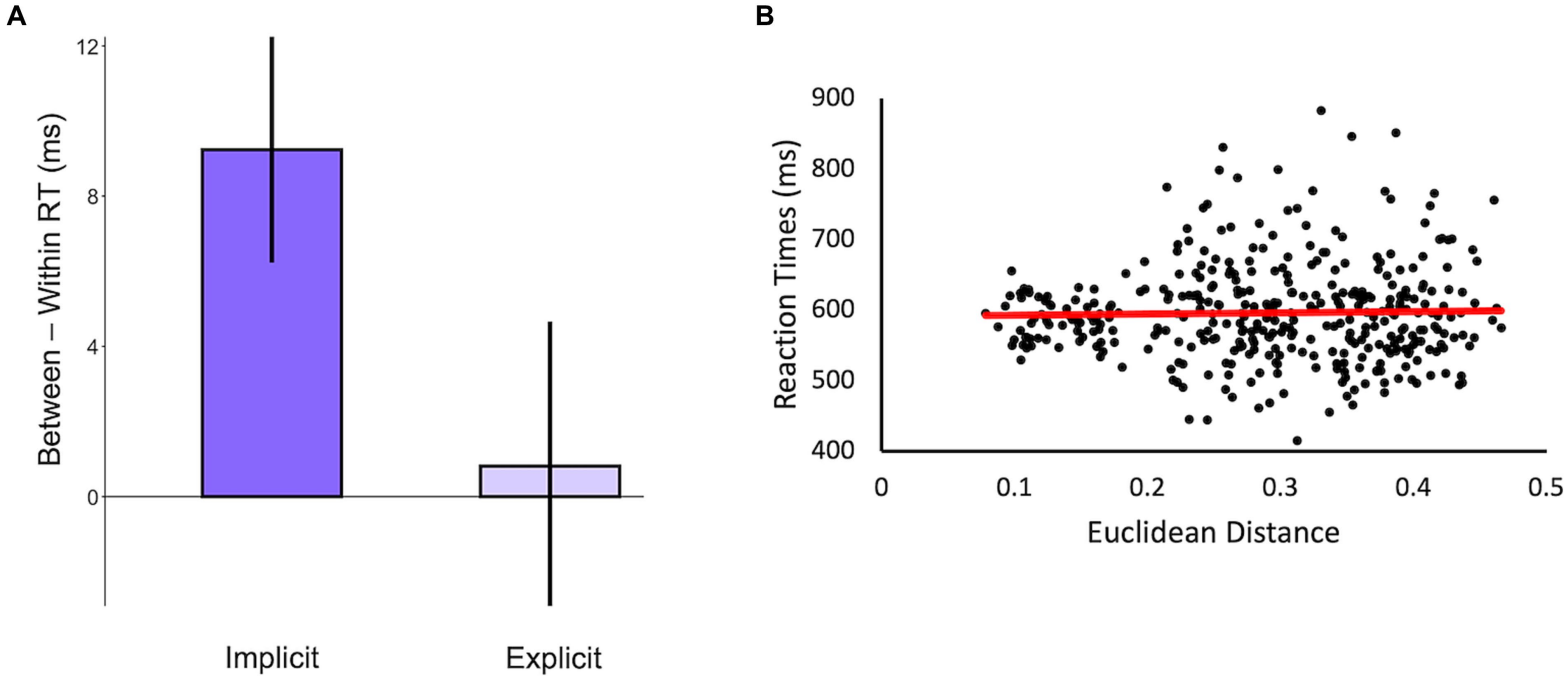
**(A)** Difference scores calculated from the in-person priming experiment based on the categories of abstract nouns identified using implicit similarity judgments (i.e., data displayed in the red bar of Figure 3B) and explicit feature ratings from the ACF database. **(B)** A scatterplot of the Euclidean distances calculated from the odd-one-out triad task and the reaction times from the priming task for all prime-probe pairs of abstract words. The correlation was not significant (*r* = 0.02, *p* = 0.65).

Next, we tested whether the significant priming effect for abstract words was specific to our method of identifying categories or if using explicit feature ratings would also suffice. To do so, we reshuffled the category membership of the words using the clusters derived from explicit feature ratings in the ACF database (Supplementary Figure 1) and calculated the magnitude of priming assuming these categories. We then compared the magnitude of priming to the priming effect found using the categories that we identified using similarity judgments about the abstract words. As predicted, we did not find a significant priming effect for abstract nouns when the categories were based on explicit feature ratings (*t*_(24)_ = 0.21, *p* = 0.84) and a repeated-measures ANOVA revealed an interaction between the priming magnitudes, such that there is less priming when categories are based on explicit feature ratings compared to when categories are based on implicit judgments of similarity (*F*_(1,24)_ = 3.74, *p* = 0.06 – Figure 4A).

Finally, we addressed the concern that abstract nouns do not fit naturally into categories but are instead organized within a continuous semantic space. In this case, the categories identified using our method might simply be an artifact of using a clustering algorithm on the similarity matrix of abstract nouns. If so, then we should expect to find that RTs for pairs of abstract nouns are correlated with the Euclidean distances between the words, regardless of whether they are from the same category. As predicted, we did not find a significant correlation between RTs and Euclidean distances across prime-probe pairs of abstract nouns (*r* = 0.02, *p* = 0.65; Figure 4B). This, combined with the replicated pattern of clustering across independent datasets and significant priming for within-category abstract words indicates that the categories identified in this study reflect intrinsic categories within the semantic space of abstract nouns.

## Discussion

We collected non-verbal and implicit judgments about which dimensions are relevant to the similarity between pairs of 50 abstract nouns and then used a rigorous clustering algorithm to identify categories within the representational space of the words. We then used automatic semantic priming to ensure that we identified intrinsic categories, rather than arbitrary categories resulting from a strategic process. We found five intuitive and replicable semantic categories within the representational space of the 50 abstract nouns and found significant automatic semantic priming for abstract nouns and a control set of concrete nouns. To the extent that our results generalize to the huge corpus of abstract words, our results suggest that the semantic space of abstract words has a category-like structure that is similar to the taxonomic structure of concrete words.

Importantly, we also found that our method of mapping the representational space of abstract words provides a significant advantage over more commonly used methods that are based on collecting explicit judgments about abstract words. In Experiment 1, we used chatGPT to show that the categories from the triad task are more easily labeled than the categories defined using dimensional weights from an explicit rating experiment (Troche et al., 2017). In Experiment 2, we showed that the significant automatic semantic priming effect that was replicated across two independent datasets is eliminated when the words are re-categorized based on the dimensions from the explicit ratings. The practice of collecting explicit judgments about (abstract and concrete) concepts – whether by asking people to list relevant features of concepts or to rate the concepts along predetermined dimensions – is ubiquitous in cognitive science (McRae et al., 2005; Crutch et al., 2013; Devereux et al., 2014; Villani et al., 2019; Lynott et al., 2020; Banks and Connell, 2023). However, this method is not ideal for characterizing the representational space of concepts because people may not always list all of the relevant features of the concepts (McRae et al., 2005; Löhr, 2023) or the experimenter may choose suboptimal feature dimensions to include in the experiment, thus leaving the representational space incomplete – this is true for concrete concepts and it is doubly true for abstract concepts since they lack easily referenced perceivable features (Borghi et al., 2017). By contrast, the odd-one-out similarity task used in Experiment 1 collects judgments of similarity between word pairs in the presence of a third word in each trial. The third word provides a context for the word pair and thus implicitly highlights the relevant dimension (s) of similarity between the word pair. For example, “sadness” and “hope” might be judged as similar when shown in a trial with “variety” because the participant chooses “variety” as the odd one out, but the words might be judged as dissimilar when shown with “worry”, since the participant might now choose “hope” as the odd one out. From these two trials, it seems that affect and valence are important dimensions underlying the semantic relation between “sadness” and “hope”. Across enough trials (and different contexts), we can map the multidimensional feature space of a set of concepts (Hebart et al., 2020; Muttenthaler et al., 2022). In the current study, we demonstrated the utility of this method by using it to identify behaviorally relevant categories in a set of abstract nouns.

To be clear, we are not claiming that results from experiments like the one used in Troche et al. (2017) are wrong, rather our results suggest that we can better derive validated categorical boundaries using the triad task. Far from thinking that collecting explicit ratings is a futile endeavor, we believe that results from such rating paradigms, and other methods used to understand word meaning, are complimentary to information gained from using our method of collecting implicit measures of similarity. For example, it would be interesting to further compare the dimensions derived from different methods and then potentially optimally combine them to better map the representational space of words. Furthermore, other information that is not directly related to conceptual content (e.g., age of acquisition, modality of acquisition, reliance on others to learn a given word) would also likely be quite valuable in future pursuits of mapping the representational space of abstract (and concrete) concepts.

The main limitation to the current study is the relatively small set of words used in the experiment. However, our decision to use a set of 50 abstract nouns was a practical one. Since the odd-one-out task had not been used with abstract concepts, we chose a small, but adequate, number of words to ensure that we could sample the entire space of triads and thus get a reliable estimate of the representational space of the abstract concepts. Indeed, we were able to collect two complete sets of triads for the 50 words so we could replicate the results across independent datasets (19,600 trials per set). Our method of replicating the category structure within this subset of abstract concepts across large groups of participants and different experimental paradigms produced results that should generalize to the full domain of abstract concepts. However, the methods in Experiment 1 were intentionally conservative to ensure that the categories were stable, thus leaving 11 words unsorted. The 11 words were unsorted here because of our decisions to include a size constraint for the categories and to require the categories to replicate across independent datasets, not necessarily because they are uncategorizable concepts – in fact, this is an empirical question that can be answered by extending our method to a larger set of abstract concepts. Thus, the results of our study demonstrate that it is a worthwhile endeavor to collect similarity judgments using the odd-one-out triad task on a larger set of abstract (and concrete) words to obtain a more complete map of the representational space that underlies our conceptual knowledge. Even prior to generalizing the results of this study to a larger set of concepts, the categories identified here can be used to select validated stimuli in future experiments.

In conclusion, we identified behaviorally valid categories within the representational space of 50 abstract nouns. To do so, we used a method of collecting implicit measures of similarity between the concepts to initially define the category structure and showed that this method provides more stable and interpretable results than commonly used methods that are based on collecting explicit judgments about abstract concepts. We then used automatic semantic priming as a strong test of the intrinsic nature of the categories. Taken together, our results demonstrate that abstract concepts can be characterized beyond their negative relation to concrete concepts by investigating their relation to one another using the appropriate methods.

## Data availability statement

The original contributions presented in the study are included in the article/Supplementary material, further inquiries can be directed to the corresponding author. The raw data used in this study are available on the Open SCience Foundation webpage. doi: 10.17605/OSF.IO/MJUKT.

## Ethics statement

The studies involving humans were approved by NIH Institutional Review Board. The studies were conducted in accordance with the local legislation and institutional requirements. The participants provided their written informed consent to participate in this study.

## Author contributions

AP: Conceptualization, Formal analysis, Investigation, Methodology, Supervision, Writing – original draft. JS: Data curation, Formal analysis, Investigation, Writing – original draft. JD: Data curation, Formal analysis, Investigation, Writing – original draft. SG: Conceptualization, Methodology, Supervision, Writing – review & editing. AM: Conceptualization, Supervision, Writing – review & editing.
