# Supplementary material for: Taxonomic structure in a set of abstract concepts
Source: Front Psychol. 2024 Jan 4;14:1278744. doi: 10.3389/fpsyg.2023.1278744 (PMC10794597; doi:10.3389/fpsyg.2023.1278744)
Supplement: Supplementary file 1 [file Image_1.pdf]

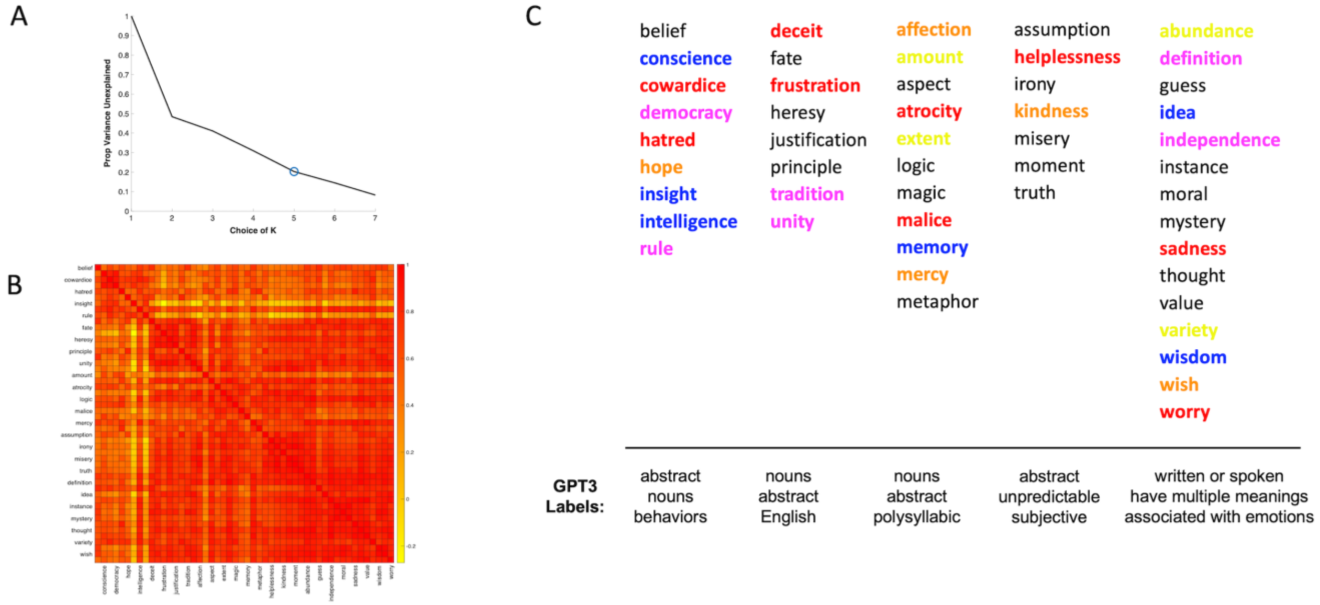

Supplemental Figure 1. A) The elbow plot for k-means clustering of the fifty abstract nouns based on explicit similarity judgments from the ACF database. The joint at k=5 is circled. B) The similarity matrix based on explicit similarity judgments. For readability, the odd rows and even columns of the matrix are labelled. C) The fifty abstract nouns separated into the five k-means clusters. The color coding depicts the category membership of the words that was identified using implicit similarity judgments in Experiment 1. Labels generated by the GPT3 model are listed at the bottom of the figure, beneath each cluster.
