# Supplementary material for: Taxonomic structure in a set of abstract concepts
Source: Front Psychol. 2024 Jan 4;14:1278744. doi: 10.3389/fpsyg.2023.1278744 (PMC10794597; doi:10.3389/fpsyg.2023.1278744)
Supplement: Supplementary file 2 [file Image_2.pdf]

Thirty concrete words

apple	cat	cottage	chisel	eagle
banana	cow	hotel	crowbar	owl
mango	dog	inn	hammer	parrot
peach	elephant	mansion	pliers	pigeon
pear	horse		screwdriver	robin
	lion		wrench	woodpecker
	pig			
	sheep			
	tiger			

Supplemental Figure 2. The thirty concrete nouns chosen from the Connecticut Category Norms database as controls for the automatic semantic priming experiment. The words are organized by category (fruit, four-legged animals, dwellings, tools, and birds).
